# Newly developed Laboratory-based Size exclusion chromatography Small-angle x-ray scattering System (La-SSS)

**DOI:** 10.1038/s41598-019-48911-w

**Published:** 2019-08-30

**Authors:** Rintaro Inoue, Tatsuo Nakagawa, Ken Morishima, Nobuhiro Sato, Aya Okuda, Reiko Urade, Rina Yogo, Saeko Yanaka, Maho Yagi-Utsumi, Koichi Kato, Kazuki Omoto, Kazuki Ito, Masaaki Sugiyama

**Affiliations:** 1 Institute for Integrated Radiation and Nuclear Science, Kyoto University, Kumatori, Sennan-gun, Osaka, 590-0494 Japan; 2 Unisoku Co. Ltd., 3-4-2 Kasugano, Hirakata City, Osaka, 573-0131 Japan; 3 Exploratory Research Center on Life and Living Systems (ExCELLS) and Institute for Molecular Science (IMS), National Institutes of Natural Sciences, 5-1 Higashiyama, Myodaiji, Okazaki, 444-8787 Japan; 4 Graduate School of Pharmaceutical Sciences, Nagoya City University, 3-1 Tanabe-dori, Mizuho-ku, Nagoya, Aichi, 467-8603 Japan; 5 Rigaku Corp. 12-9-3 Matsubara, Akishima City, Tokyo, 196-8666 Japan

**Keywords:** Characterization and analytical techniques, Nanoscale biophysics

## Abstract

To understand a biological system, it is important to observe structures of biomolecules in the solution where the system is functionalized. Small-Angle X-ray Scattering coupled with Size Exclusion Chromatography (SEC-SAXS) is one of techniques to selectively observe the target molecules in the multi-component system. However, this technique is believed to be available only with a synchrotron-based SAXS instrument due to requirement of high beam intensity and, therefore, the limitation of the beam time was obstacle to satisfy demands from many bio-researchers. We newly developed Laboratory-based Size exclusion chromatography SAXS System (La-SSS) by utilizing a latest laboratory-based SAXS instrument and finely optimization of the balance between flow rate, cell volume, irradiation time and so on. La-SSS succeeded not only decoupling of target protein(s) from non-specific aggregates but also measurement of each concerned component in a multi-component system. In addition, an option: “stopping mode”, which is designed for improving statistics of SAXS profile, realized a high S/N data acquisition for the most interesting protein in a multi-component system. Furthermore, by utilizing a column having small bed volume, the small-scale SEC-SAXS study makes available. Through optimization of instrumental parameters and environments, La-SSS is highly applicable for experimental requirements from various biological samples. It is strongly expected that a La-SSS concept must be a normal option for laboratory-based SAXS in the near future.

## Introduction

Small-angle X-ray scattering (SAXS) offers the overwhelming opportunities for structural analysis on various biological samples in a solution. However, SAXS has an inherent difficulty: only a subtle contamination of non-specific aggregate(s) produces upturn at the low *Q* region since a forward scattering intensity (*I*(0)) is almost proportional to the square of molecular weight (*M*_w_) of a particle. As a result, it makes difficult to evaluate *I*(0) and radius of gyration (*R*_g_) of the non-aggregated target protein when the small amount of non-specific aggregates are contaminated in a sample solution. In other words, the elimination of unfavourable aggregated components from the solution is essential for success of the structural analysis in a SAXS study.

Size exclusion chromatography (SEC) is one of standard methods for the separation of a concerned component in a multi-component system through the difference in sizes of components. Then, it is a usual procedure that the separation of the concerned component from the unfavourable aggregates with SEC prior to SAXS measurements. In the progress of this concept, it is becoming a standard method to measure SAXS by directly injecting an eluted solution from SEC column into a SAXS cell: this is called, “SEC-SAXS”. With the SEC-SAXS technique, we can measure the SAXS of the just separated component prior to its re-aggregation.

SEC-SAXS was considered to be realized only with a synchrotron-based SAXS instrument due to requirement of higher beam intensity by using a flow measuring system. Therefore, the first SEC-SAXS was developed by David and Pérez^1^ at the SOLEIL. After their pioneering work, the SEC-SAXS technique has been widely spread over many synchrotron facilities^2–6^ around the world. On the other hand, it is still difficult that every biologist performs a SEC-SAXS experiment since the beam time at synchrotron facilities is quite limited. The shortage of beam time should be solved by realization of *a laboratory-based SEC-SAXS*. Here raises a question, “Is it possible to construct a laboratory-based SEC-SAXS”? even though the most people *a priori* believe that SEC-SAXS is an only available technique at synchrotron facilities.

Equipment for laboratory-based SAXS, such as a focusing mirror, a collimation system with less parasite scattering pinholes and a photon-counting detector, is in distinguished progress. As a result, a photon flux at sample position in a latest laboratory-based SAXS reaches as much as 1/100 compared to those in a synchrotron-based SAXS instrument^7^. Under this situation, Bucciarelli *et al*.^8^ launched a laboratory-based SEC-SAXS system and succeeded to obtain the SAXS data, which enables the further analysis of a non-aggregated target component for the first time. Here, at least three technical improvements must be considered for a next generation laboratory-based SEC-SAXS. The first one, which is the nuisance problem in SEC-SAXS measurement, is re-mixing between the separated samples. This problem is mainly originated from the retention of samples at a SAXS cell. The proteins with the same sizes are eluted out from a SEC column as groups from larger to smaller ones in the time order. Therefore, the longer retention of the group in the cell has a possibility to make the mixture of the next coming group with the different size. This re-mixing results in the measured SAXS profile that includes the scatterings from the different proteins. The re-mixing is observed as broadening of time evolution of protein concentration at the SAXS cell and, in the worst case, the elution peaks of proteins having different sizes would be overlapped in the SEC-elution chart. We dare to name this re-mixing problem as “broadening problem”. The broadening could make it difficult to selectively perform a structural analysis on an interested component in a multi-component complex system. Therefore, the elimination of “broadening problem” in SEC-SAXS is the first technical requirement. The second point is the reduction of injection sample amount, which offers the chance for SEC-SAXS measurements on the sample with a little of available amount. The third point is the amendment of counting statistics of SAXS profiles, contributing to making the quality of structure analysis higher.

We firstly constructed the SEC-SAXS system, which is free from the broadening problem even at slow flow rate. Secondly, we introduced an additional technique for enhancing the data quality and, finally, reduced sample injection volume. In this manuscript, we reported the feasibility of our finely optimized Laboratory-based SEC-SAXS System (La-SSS) to several proteins.

## Results and Discussion

### Detail of setup of La-SSS

We have to take into consideration of the balance between a flow rate and separation resolution, keeping the statistics for data analysis. Namely, the faster flow rate reduces the re-mixing by washing the sample solution out from the SAXS cell quickly, however might deteriorate the separation resolution and the statistics of scattering data due to shortening of an exposure time. Firstly, to figure out the required exposure time experimentally, an ovalbumin (OVA) solution with the concentration of 1.5 mg/mL, which is the nominal protein concentration on a standard SAXS measurement, was measured with the SAXS instrument, NANOPIX (see Materials and methods: SAXS instruments). It was estimated that measurement time of c.a. 20 minutes was at least necessary for the determination of reliable *R*_g_ value. Next, we optimized the flow rate with Superdex 200 increase 10/300 GL. Under the conventional flow rate of 0.50 mL/min, the full width at half maximum of elution peak of monomeric OVA was found to be 1.0 min. Accordingly, the flow rate for La-SSS should be slower than 0.025 (=0.50/20) mL/min to satisfy the required exposure time. Considering the above experimental requirements, the standard flow rate was selected to 0.020 mL/min for La-SSS. Under this flow rate, the flowing volume corresponds to the SAXS cell (40 *μ*L) in 2 minutes. It implies that the sample at X-ray irradiation area will be thoroughly washed out within 2 minutes. Namely, La-SSS is in principle expected to be free from “broadening problem”. In the next section, we will verify the absence of “broadening problem” in more detail based on experimental results.

**Figure 1:**
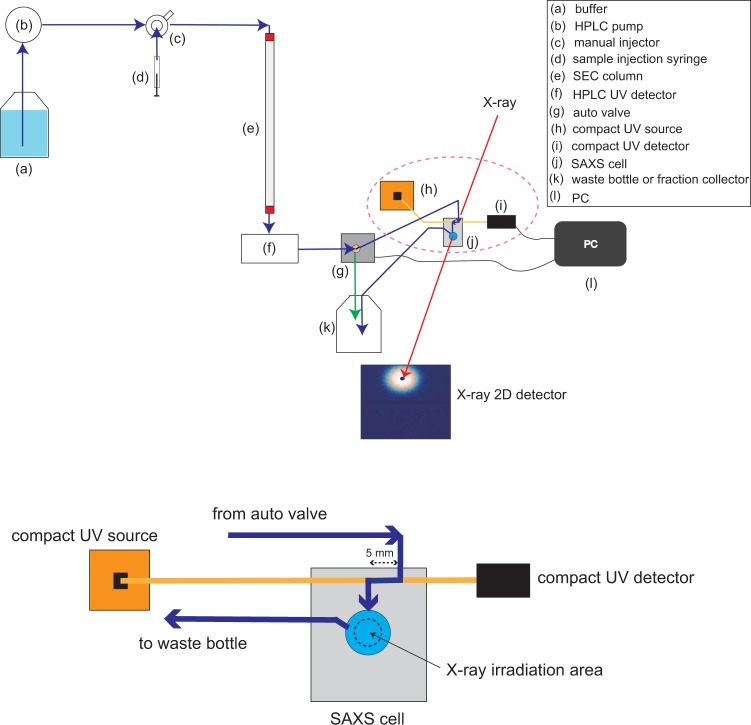
Schematic overview of La-SSS, which consists of (**a**) buffer bottle, (**b**) HPLC pump, (**c**) manual injector, (**d**) sample injection syringe, (**e**) SEC column, (**f**) HPLC UV detector (**g**) auto valve, (**h**) compact UV source, (**i**) compact UV detector, (**j**) SAXS cell, (**k**) waste bottle or fraction collector, and (**l**) PC that is used for monitoring UV intensity at SAXS cell position and for switching the flow path between a waste bottle and a SAXS cell. The down figure corresponds to the magnified pictures of SAXS cell, compact UV source, and compact UV detector, which are highlighted by pink dotted circle in upper figure. Dark blue and yellow lines correspond to a flow path and an optical fiber, respectively, and the sample flow path was set to 5 mm. Black dotted circle indicates X-ray irradiation zone. The system has another function “stopping mode”. When the OD_280_ value exceeds the set value, the auto valve automatically switches the path from a SAXS cell to a waste bottle, as shown by green arrow. As a result, the solution exceeding the set OD_280_ value, target protein solution, remains in the SAXS cell.

The detail of components of our La-SSS is shown in Table S1 (refer to supplementary materials) and the schematic view of our La-SSS is also shown in Fig. 1.

### Absence of “broadening problem” at SAXS cell

**Figure 2:**
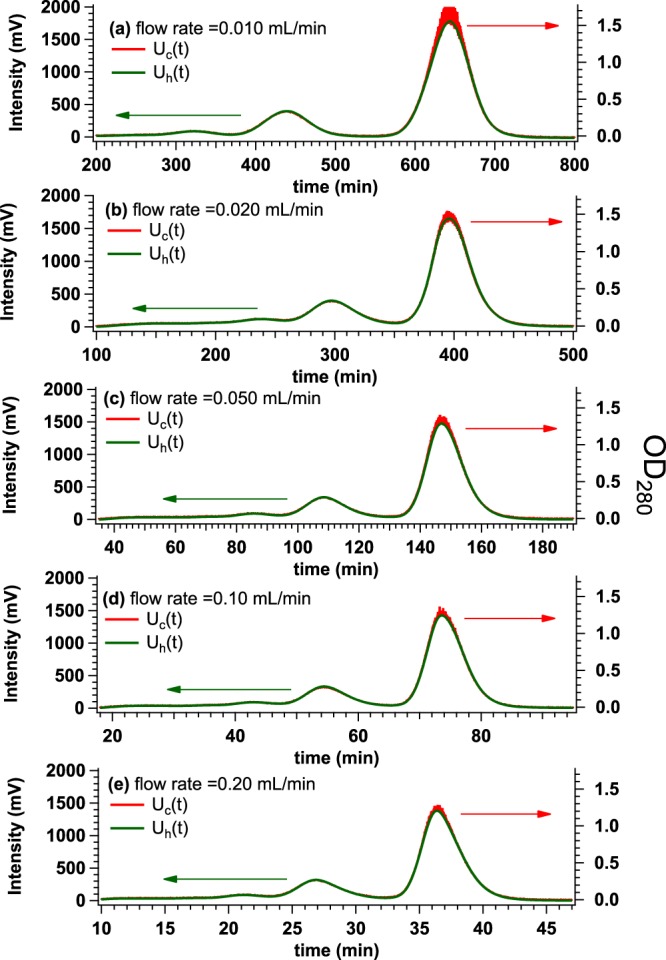
Time evolutions of UV intensity at 280 nm from UV-HPLC (green line) (*U*_h_(*t*)) and OD_280_ from UV-SAXS-cell (*U*_c_(*t*)) (red line) at the flow rate of (**a**) 0.010 mL/min, (**b**) 0.020 mL/min, (**c**) 0.050 mL/min, (**d**) 0.10 mL/min and (**e**) 0.20 mL/min. In all experiments, BSA solutions were applied with the initial concentration and the volume of 5.4 mg/mL and 500 *μ*l, respectively.

**Figure 3:**
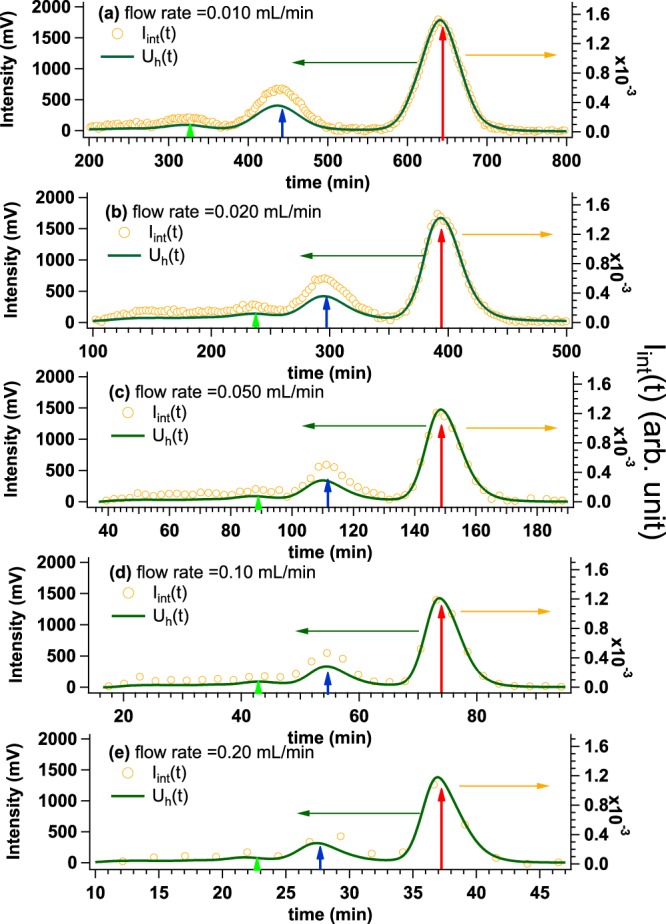
Time evolutions of UV intensity at 280 nm from HPLC (green line) (*U*_h_(*t*)) and SAXS integrated intensity (*I*_int_(*t*)) (yellow circle) at the flow rate of (**a**) 0.010 mL/min, (**b**) 0.020 mL/min, (**c**) 0.050 mL/min, (**d**) 0.10 mL/min and (**e**) 0.20 mL/min obtained by injecting BSA solution with the initial concentration and the volume of 5.4 mg/mL and 500 *μ*l, respectively. Three peaks were observed from UV-HPLC, of which peak positions are highlighted by light green, blue and red arrows from the higher molecular weight component to the lower one.

It was reported that the slower flow rate is beneficial for the improvement of separation resolution of SEC^9^. We then experimentally try to verify that La-SSS is free from the broadening problem at several flow rates (including our standard flow rate, 0.020 mL/min) with bovine serum albumin (BSA) solution: the initial concentration and injection volume of 5.4 mg/mL and 500 *μ*l, respectively. Firstly, by changing the flow rates from 0.010 mL/min to 0.20 mL/min, we have compared the time evolution of UV intensity at exit of HPLC (*U*_h_(*t*): *t* is elution time) to that at just upstream of SAXS-cell (*U*_c_(*t*)) (see Fig. 1(f,h,i), respectively). As shown in Fig. 2, it was confirmed that *U*_c_(*t*) nicely coincided with *U*_h_(*t*) in all the flow rates. Next, we also have compared *U*_h_(*t*) to the time evolution of SAXS intensity integrated from 0.015 Å^−1^ to 0.030 Å^−1^ (*I*_int_(*t*)). As shown in Fig. 3, regardless of flow rate, three peaks were observed in *U*_h_(*t*). Their peak positions are highlighted by light green, blue and red arrows from the high molecular weight component to the low one. *I*_int_(*t*) of the peak exhibiting the highest intensity nicely reproduced *U*_h_(*t*) without broadening at all the flow rates investigated. It is then concluded that the SAXS cell in La-SSS is essentially free from “broadening problem” even at the slowest flow rate (0.010 mL/min). It should also be noted that good separation of three peaks is observable from *I*_int_(*t*) at the flow rate of 0.010 mL/min and 0.020 mL/min.

### SEC-SAXS measurements: Observation of protein with the relatively low molecular weight including the aggregates in solution

**Figure 4:**
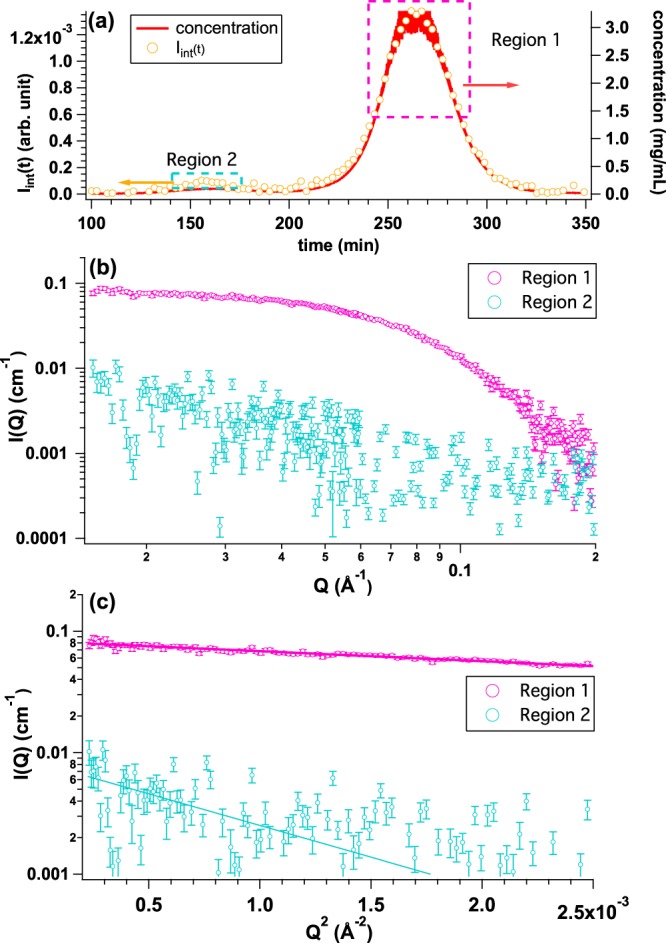
(**a**) Time evolutions of integrated SAXS intensity (*I*_int_(*t*)) (yellow circle) and concentration calculated from *U*_c_(*t*) (UV-SAXS-cell: red line) by injecting OVA solution with the initial concentration and the volume of 5.0 mg/mL and 500 *μ*l, respectively. We selected two regions; Region 1 (peak at *t* = 265 min: highlighted by pink dotted rectangle) and Region 2 (peak at *t* = 155 min: highlighted by light blue rectangle) for obtaining averaged SAXS profiles. (**b**) Averaged SAXS profiles over Region 1 (pink circle) and Region 2 (light blue circle), respectively. (**c**) Guinier plots of averaged SAXS profiles from Region 1 (pink, *R*_g_ = 23.9 ± 0.4 Å) and Region 2 (light blue, 63.8 ± 28.4 Å), respectively.

We tested the performance of La-SSS with relatively low molecular weight protein: OVA with *M*_w_ = 45 kDa. OVA solution was injected to SEC column with the initial concentration and volume of 5.0 mg/mL and 500 *μ*l, respectively. The magnitude of scattering vector: *Q* is given by 4*π*sin(θ)/λ, where 2θ and λ correspond to scattering angle and wavelength of X-ray, respectively. Figure 4(a) shows *I*_int_(*t*) (yellow circle) and the time evolution of protein concentration (red curve) calculated from *U*_c_(*t*). There are two peaks in the elution chart: one is a clear and extinct peak at 265 min and the other is a weak peak at 155 min. We then averaged the SAXS profiles at two regions; Region 1 (peak at *t* = 265 min: highlighted by pink dotted rectangle) and Region 2 (peak at *t* = 155 min: highlighted by light blue dotted rectangle), as shown in Fig. 4(a). The averaged SAXS profiles and their Guinier plots from Regions 1 and 2 are shown in Fig. 4(b,c), respectively. *R*_g_ from Region 1 is found to be 23.9 ± 0.4 Å, which is well consistent with that of monomeric OVA (*R*_g_ = 23.9 ± 0.2 Å) measured with synchrotron-based SAXS instrument^4^. Although the statistics of the averaged SAXS profile from Region 2 is not good due to the quite low protein concentration (~0.07 mg/mL), *R*_g_ value was calculated to be 63.8 ± 28.4 Å. It is considered that Region 2 is dominated by aggregated OVA. In conclusion, monomeric OVA was well separated from the aggregates by La-SSS.

Here, we would like to show the ability of La-SSS to detect the shape difference of protein having similar molecular weight. We selected Fc portion of immunoglobulin G^10^ with molecular weight of 50 kDa, which is similar with OVA (45 kDa). Concerning about shape, Fc has a horseshoe-like shape, whereas OVA possesses a barrel-like one. We performed SEC-SAXS measurement of Fc with La-SSS by injecting the concentration and volume of 5.0 mg/mL and 500 *μ*l, respectively. The scattering profiles from monomeric OVA and Fc are shown in Fig. S1(a). Calculated *R*_g_ value of Fc was 27.4 ± 0.4 Å, which is relatively different from that of OVA (23.9 ± 0.4 Å). Utilizing the same *Q* range for OVA solution, we also calculated distance distribution function (*P*(*R*) function) of Fc. *P*(*R*) functions of Fc and OVA are plotted in Fig. S1(b). It can be clearly seen the difference in *P*(*R*) functions. *R*_g_ and *P*(*R*) function reflect the difference in shape between two proteins. In conclusion, with La-SSS, we can detect the shape difference as well.

### SEC-SAXS measurement: Observation of protein with the higher molecular weight including the aggregates in solution

**Figure 5:**
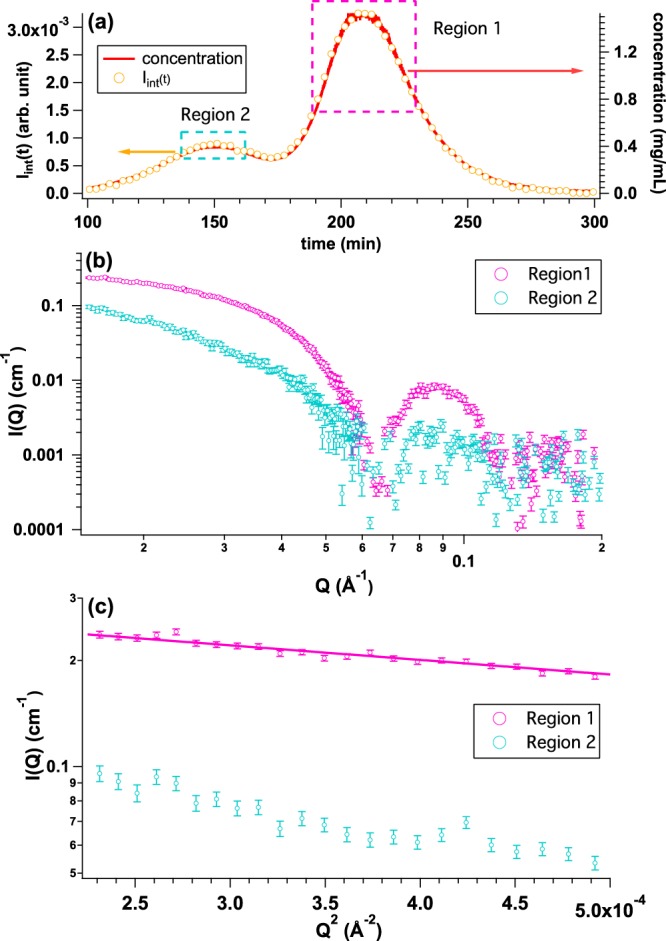
(**a**) Time evolutions of integrated SAXS intensity (*I*_int_(*t*)) (yellow circle) and concentration calculated from *U*_c_(*t*) (UV-SAXS-cell: red line) by injecting AF solution with the initial concentration and the volume of 5.1 mg/mL and 500 *μ*l, respectively. Two peaks were observed in both of *I*_int_(*t*) and *U*_c_(*t*). Main and sub peaks were located at 210 min and 150 min, respectively. We selected two regions; Region 1 (peak at *t* = 210 min: highlighted by pink dotted rectangle) and Region 2 (peak at *t* = 150 min: highlighted by light blue dotted rectangle) for obtaining averaged SAXS profiles. (**b**) Averaged SAXS profiles from Region 1 (pink circle) and Region 2 (light blue circle), respectively. (**c**) Guinier plots of averaged SAXS profiles from Region 1 (pink*, R*_g_ = 53.2 ± 1.6 Å) and Region 2 (light blue, *R*_g_ = N. A.), respectively.

We also checked the performance of La-SSS with apoferritin (AF) as a relatively higher molecular weight protein (440 kDa). AF solution with the initial concentration and volume of 5.1 mg/mL and 500 *μ*l, respectively, was injected to SEC column. Figure 5(a) shows *I*_int_(*t*) (yellow circle) and the time evolution of protein concentration (red curve). Two peaks were observed in *I*_int_(*t*) and the time evolution of concentration. Main and minor peaks were located at 210 min and 150 min, respectively. We then averaged the SAXS profiles at two regions; Region 1 (peak at *t* = 210 min: highlighted by pink dotted rectangle) and Region 2 (peak at *t* = 150 min: highlighted by light blue dotted rectangle), as shown in Fig. 5(a). The averaged SAXS profiles and their Guinier plots are shown in Fig. 5(b,c), respectively. A clear upturn at low *Q*-region is observed in the SAXS profile from Region 2. *R*_g_ from Region 1 is found to be 53.2 ± 1.6 Å, which is nicely consistent with *R*_g_ reported by Zabelskii *et al*.^11^ as that of a monomer of AF (*R*_g_ = 52.9 Å). On the other hand, *R*_g_ value satisfying the relationship in *Q* < 1.3/*R*_g_ was not obtained from Region 2 due to too large *R*_g_ value. It is again confirmed that La-SSS also contributes to the separation of native protein complex from aggregated one for the protein with higher molecular weight.

### Improvement of statistics: stopping mode

**Figure 6:**
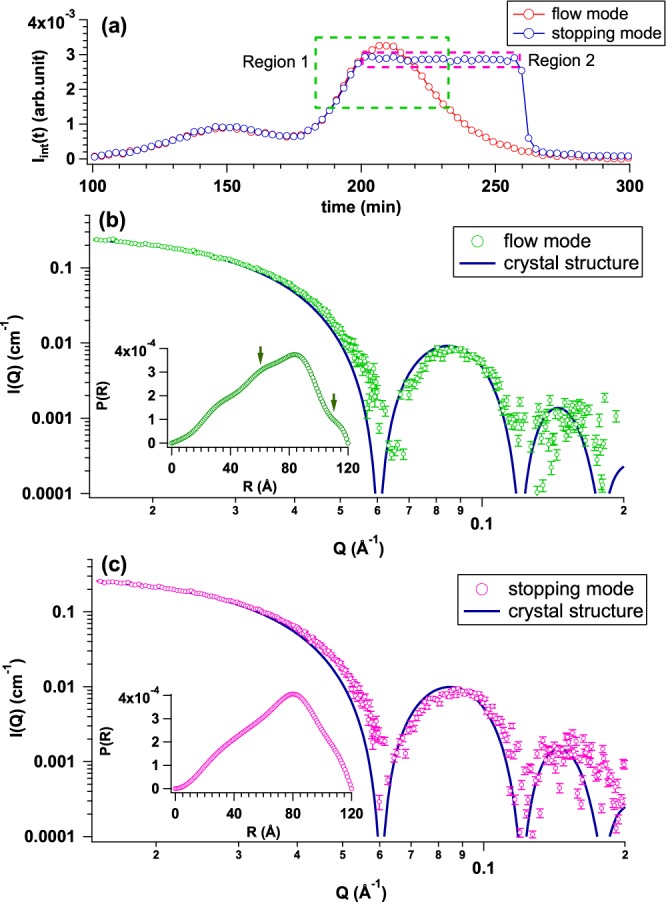
(**a**) Time evolutions of integrated SAXS intensity (*I*_int_(*t*)) from flow mode (red circle) and stopping mode (blue circle) by applying AF solution with the initial concentration and the volume of 5.1 mg/mL and 500 *μ*l, respectively. We selected two regions; Region 1 (highlighted by green dotted rectangle) and Region 2 (highlighted by pink dotted rectangle) for obtaining averaged SAXS profiles. (**b**) Averaged SAXS profile from the Region 1 (green circle) in (**a**) and calculated SAXS profile based on crystal structure (blue curve). Inset shows *P*(*R*) calculated from averaged SAXS profile. *P*(*R*) from the flow mode is slightly oscillating due to insufficient data quality, as shown by green solid arrows. (**c**) Averaged SAXS profile from the Region 2 (pink circle) in (**a**) and calculated SAXS profile based on crystal structure (blue curve). Inset figures show *P*(*R*) functions calculated from averaged SAXS profiles.

It was confirmed that La-SSS could evaluate *R*_g_ from monomeric protein or native protein complex including aggregates. On the other hand, the recent Bio-SAXS analysis requires high statistics data in wide-*Q* region to build the three-dimensional structural model. To improve the data statistics, especially quality at high *Q*-region, we installed an optional measurement mode for La-SSS: *stopping mode*. When the OD_280_ value exceeds the set value, the flow path automatically switches to a waste bottle (see Fig. 1(k)) and the solution exceeding the set OD_280_ value remains in the SAXS cell. Figure 6 shows the performance of the stopping mode, *I*_int_(*t*)s from the flow mode (red circle) and the stopping mode (blue circle), respectively. The averaged SAXS profiles from the flow and stopping modes are shown in Fig. 6(b,c), respectively: the blue line is the scattering profile calculated with its crystal structure. The evaluated *R*_g_ values from the flow and stopping modes are 53.2 ± 1.6 Å and 53.1 ± 1.5 Å, respectively. In addition, the statistics around second fringe (*Q*~0.15 Å^−1^) is clearly improved with the aid of stopping mode. *P*(*R*) calculated by GNOM^12^ are shown in the insets of Fig. 6(b,c), respectively. *P*(*R*) from the flow mode was slightly oscillating due to insufficient data quality, as shown by solid arrows in the inset of Fig. 6(b). Contrary to this, *P*(*R*) from the stopping mode exhibited smooth curve in the whole *R* range. It is expected that this stopping mode will contribute to the improvement of statistics at the high *Q* region even with a lower concentration sample. Finally, it should be kept in mind that SAXS integrated intensity is quite sensitive to the onset of aggregation, hence the presence or absence of aggregation is always monitored by *I*_int_(*t*). In this AF case, no trace of aggregation was observed during the stopping mode for 60 minutes.

### SEC-SAXS measurements with multi-component system: BSA

**Figure 7:**
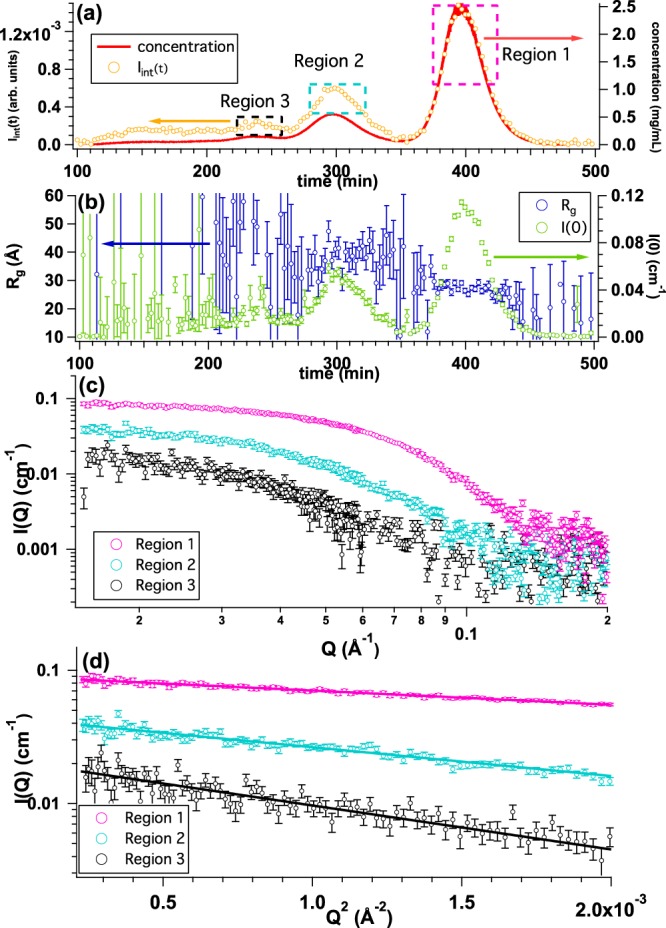
(**a**) Time evolutions of integrated SAXS intensity (*I*_int_(*t*)) (yellow circle) and concentration calculated from *U*_c_(*t*) (UV-SAXS-cell: red line) by injecting BSA solution with the initial concentration and the volume of 5.4 mg/mL and 500 *μ*l, respectively. We selected three different regions: Region 1 (peak at *t* = 400 min: highlighted by pink dotted rectangle), Region 2 (peak at *t* = 300 min: highlighted by light blue dotted rectangle) and Region 3 (peak at *t* = 240 min: highlighted by black dotted rectangle) for obtaining averaged SAXS profiles. (**b**) Time evolutions of *R*_g_ (blue circle) and *I*(0) (green circle). (**c**) Averaged SAXS profiles from Region 1 (pink circle), Region 2 (light blue circle) and Region 3 (black circle), respectively. (**d**) Guinier plots of averaged SAXS profiles from Region 1(pink, *R*_g_ = 27.3 ± 0.3 Å), Region 2 (light blue, *R*_g_ = 39.0 ± 1.9 Å) and Region 3 (black, *R*_g_ = 47.2 ± 8.0 Å), respectively.

For further exhibiting the performance of La-SSS, we also utilized La-SSS for more complex system, BSA, which is prone to form several oligomers. Prior to SEC-SAXS measurement, the AUC measurement of BSA solution was performed with the same initial concentration as that of SEC-SAXS. As shown in Fig. S2, there were three peaks in the sedimentation coefficient (*S*_20_) distribution (c(*S*_20_)): one major peak at around *S*_20_ = 4, and two minor peaks around *S*_20_ = 6 and 8. Through the relationship between *S*_20_ value and molecular weight, it was found that the components of *S*_20_ = 4, *S*_20_ = 6 and *S*_20_ = 8 correspond to monomeric dimeric and trimeric BSA oligomers, respectively. With this polydispersed BSA solution, we performed SEC-SAXS measurement. Figure 7(a) shows the time evolution of concentration (red curve) and *I*_int_(*t*) (yellow circle). Three peaks are observable from Fig. 7(a) as expected from the AUC measurement. *I*(0) and *R*_g_ as a function of time are sequentially analyzed and the results are summarized in Fig. 7(b). We then averaged the SAXS profiles at three different regions: Region 1 (peak at *t* = 400 min: highlighted by pink dotted rectangle), Region 2 (peak at *t* = 300 min: highlighted by light blue dotted rectangle) and Region 3 (peak at *t* = 240 min: highlighted by black dotted rectangle) as shown in Fig. 7(a). The averaged SAXS profiles from Regions 1, 2 and 3 are shown in Fig. 7(c) and the corresponding Guinier plots are also summarized in Fig. 7(d). The evaluated *R*_g_ values from Regions 1, 2 and 3 are 27.3 ± 0.3 Å, 39.0 ± 1.9 Å and 47.2 ± 8.0 Å, respectively. The reported *R*_g_ values of monomeric and dimeric BSA were 27.0 ± 0.1 Å and 39.0 ± 0.2 Å^13^, respectively. Hence, our evaluated *R*_g_ values were surprisingly coincided with those from the synchrotron-based SEC-SAXS studies within the experimental errors. Judging from the AUC result, it is supposed that *R*_g_ = 47.2 ± 8.0 Å corresponds to trimer of BSA. It is also considered that La-SSS is applicable for the multi-component system.

### SEC-SAXS measurements: Observation of protein with the low molecular weight: PNGase-PUB

**Figure 8:**
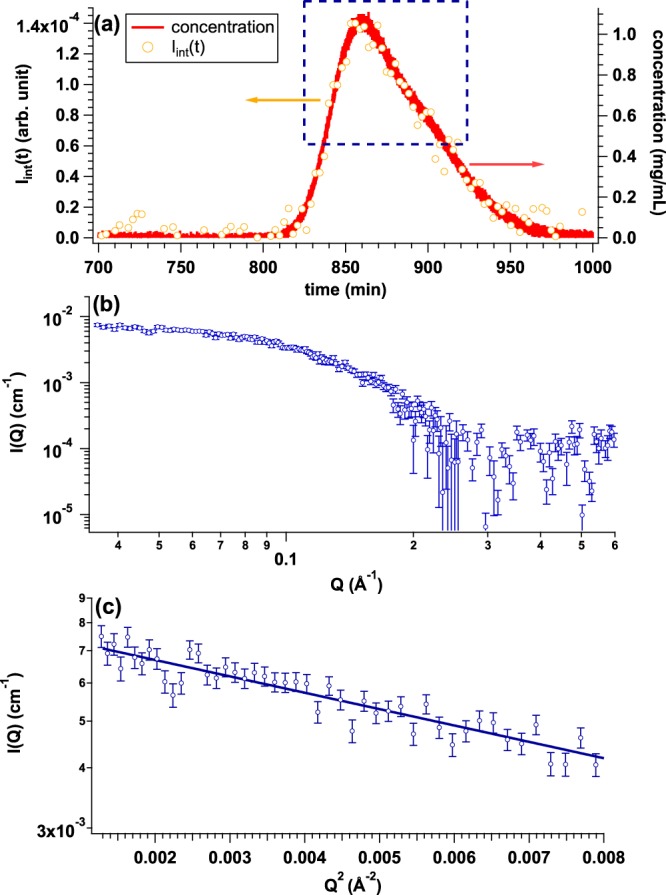
(**a**) Time evolutions of integrated SAXS intensity (*I*_int_(*t*)) (yellow circle) and concentration calculated from *U*_c_(*t*) (UV-SAXS-cell: red line) by injecting PNGase-PUB solution with the initial concentration and the volume of 2.7 mg/mL and 300 *μ*l, respectively. We selected a region at *t* = 860 min highlighted by blue dotted rectangle for obtaining averaged SAXS profile. (**b**) Averaged SAXS profile from the region highlighted by blue dotted rectangle in (**a**). (**c**) Guinier plot of averaged SAXS profile (*R*_g_ = 15.3 ± 0.5 Å).

From the SEC-SAXS studies on OVA, Fc, AF and BSA, we could successfully exhibit the performance of La-SSS. In order to extend the accessible range of *M*_w_ of target protein, we also tested the performance of La-SSS with low molecular weight protein by changing sample to detector distance. The target protein is PNGase-PUB^14^ with *M*_w_ = 13.4 kDa. For this SEC-SAXS study, Superdex 75 increase 10/300 GL was utilized for SEC column. PNGase-PUB solution was injected to SEC column with the initial concentration and volume of 2.7 mg/mL and 300 *μ*l, respectively. In order to enhance S/N of SAXS data, the flow rate of 0.010 mL/min was selected for this study. Figure 8(a) shows *I*_int_(*t*) integrated from 0.035 Å^−1^ to 0.050 Å^−1^ (yellow circle) and the time evolution of protein concentration (red curve) calculated from *U*_c_(*t*). We then averaged the SAXS profile at *t* = 860 min: highlighted by blue dotted rectangle. The averaged SAXS profile and its Guinier plot are shown in Fig. 8(b,c), respectively. *R*_g_ was calculated to 15.3 ± 0.5 Å, which is consistent with that calculated from its crystal structure (=15.8 Å) within the experimental error. Present result surely certificates the applicability of La-SSS to low molecular weight protein as well.

### Further challenge: Approach for small-scale SEC-SAXS system

**Figure 9:**
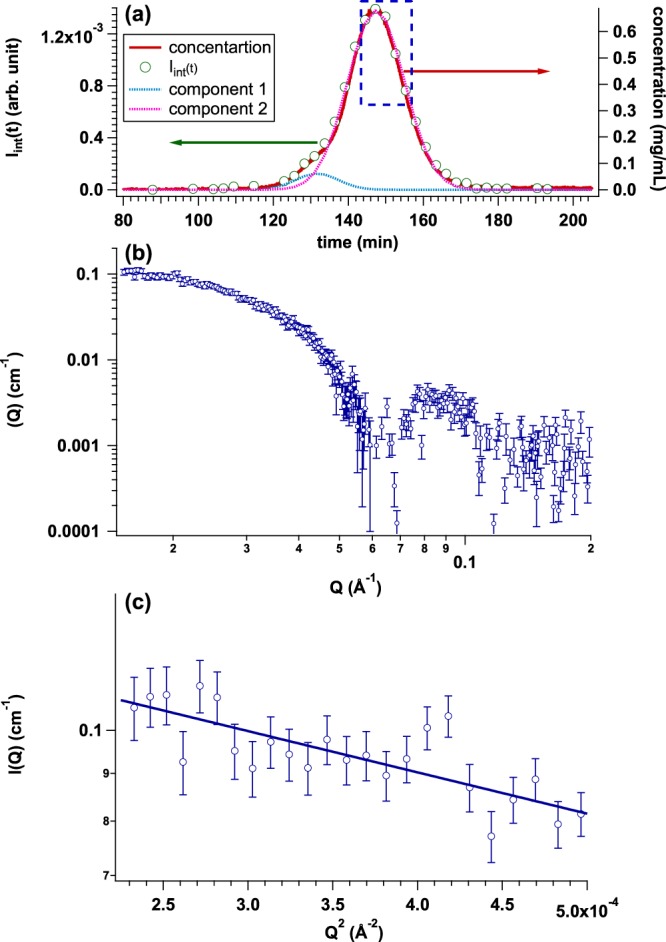
(**a**) Time evolutions of integrated SAXS intensity (*I*_int_(*t*)) (green circle) and concentration calculated from *U*_c_(*t*) (UV-SAXS-cell: red line) by injecting AF solution with the initial concentration and the volume of 5.1 mg/mL and 50 *μ*l, respectively. Two Gaussians are used for the description of *I*_int_(*t*) and dotted curve corresponds to each component: light blue (component 1) and pink (component 2), respectively. (**b**) Averaged SAXS profile from the region highlighted by blue dotted rectangle in Fig. 8(a,c) Guinier plot of averaged SAXS profile (*R*_g_ = 55.2 ± 3.8 Å).

It is required for c.a. 2.5 mg (=5.1 mg/mL × 500 *μ*l) proteins to maximize the concentration of a target protein in our La-SSS (a normal scale system). However, the preparation of such huge amount is still drawback for performing a SEC-SAXS experiment. The reduction of sample amount opens the accessibility of SEC-SAXS studies, hence we also build a small-scale SEC-SAXS system: The SEC column is Superdex 200 increase 3.2/300 of which injection volume can be reduced to 50 *μ*l and the flow rate is adopted at 0.010 mL/min. We tested this small-scale SEC-SAXS system using an AF solution with the initial concentration of 5.1 mg/mL. Figure 9(a) shows the time evolution of protein concentration (red curve) and *I*_int_(*t*) integrated from 0.015 Å^−1^ to 0.030 Å^−1^ (green circle). In the small scale SEC-SAXS system, the contribution of non-natively aggregated AF was not well decoupled from that of native AF. It is expected that the resolution of separation of Superdex 200 increase 3.2/300 is not superior as that of Superdex 200 increase 10/300 GL. With the result of the small-scale SEC-SAXS system, to eliminate the contribution of non-natively aggregated AF, we firstly fitted the *I*_int_(*t*) with two Gaussians. Each Gaussian component is depicted by dotted light blue line (component 1) and dotted light pink line (component 2), respectively, in Fig. 9(a). Then, the region excluding the tail of component 1 was chosen for averaging region, as shown by blue dotted rectangle in Fig. 9(a). The averaged SAXS profile and its Guinier plot are shown in Fig. 9(b,c), respectively. *R*_g_ value was 55.2 ± 3.8 Å same as that obtained from the normal-scale SEC-SAXS system within experimental error (Fig. 5(c) and more detailed information should be referred to Table 1).

**Table 1:** Structural parameters of the studied proteins from La-SSS.

Sample	*R*_g_ (Å)	*c* (mg/mL)	*I*(0)/*c* (cm^−1^/(mg/mL))	*M*_w_ (kDa)	*D*_max_ (Å)
OVA monomer	23.9 ± 0.4	2.42	0.034 ± 0.004	59.4 ± 7.0	63
OVA aggregate	63.8 ± 28.4	0.06	0.13 ± 0.08	227.2 ± 139.8	N. A.
BSA monomer	27.3 ± 0.3	2.01	0.045 ± 0.004	70.7 ± 6.3	78
BSA dimer	39.0 ± 1.9	0.46	0.097 ± 0.01	152.4 ± 15.7	105
BSA trimer	47.2 ± 8.0	0.14	0.15 ± 0.02	235.7 ± 31.4	N. A.
Apoferritin monomer	53.2 ± 1.6	1.25	0.24 ± 0.03	393.7 ± 49.2	120
Apoferritin aggregate	N. A.	0.36	N. A.	N. A.	N. A.
PNGase-PUB monomer	15.3 ± 0.5	0.79	0.0099 ± 0.0002	13.9 ± 0.3	40
Apoferritin monomer (small column)	55.2 ± 3.8	0.57	0.24 ± 0.04	393.7 ± 65.6	N. A.

Although the error of *R*_g_ from the small-scale system is still larger than that from the normal-scale system, it is considered that the scattering profiles having similar S/N can be obtained from this small column through further optimization of experimental conditions and/or utilization of “stopping mode”. It is also expected that optimum selection of carrier of SEC column would further improve the performance of SEC-SAXS with small column volume. Finally, it will open further opportunity for La-SSS studies on small sample volume.

### Summary

In this paper, we have extended the feasibility of laboratory-based SEC-SAXS system (La-SSS) by optimizing instrumental parameters and environments. We could successfully obtain the scattering profiles and evaluate *R*_g_ of target proteins without aggregation: The relationship between data quality and flow rate is given in Fig. S3. In addition, dimensionless Kratky plot from all the monomeric proteins exhibited the bell-shaped curves having the peaks at *QR*_g_ = 1.7. These results strongly support that all the monomeric protein possesses well-folded globular structure with La-SSS (refer to Fig. S4). In addition to decoupling the target monomeric or native protein complex from non-specific aggregation, selective structural analysis of aggregates was practically realized. It is strongly expected that present performance of La-SSS must be quite effective for the system in which association/dissociation process exists. By utilizing the stopping mode, high S/N scattering curve covering high *Q* region is obtainable. Furthermore, the column having smaller bed volume supports the small volume scale SEC-SAXS measurement with La-SSS.

It is then concluded that La-SSS must be normal option for laboratory-based SAXS in near future. In addition, the concept of “stopping mode” offers the possibility to apply a Small-Angle Scattering (SAS) instruments with the lower beam intensity such as small-angle *neutron* scattering.

## Materials and Methods

### SAXS instrument

A SAXS instrument for La-SSS adopts NANOPIX (Rigaku). In NANOPIX, X-rays from a high-brilliance point-focused generator (MicroMAX-007 HFMR) were focused with a confocal multilayer mirror (OptiSAXS) and then two pinholes collimation system with the lower parasitic scattering,”ClearPinhole”, supplied for the X-rays with the flux of 2.0 × 10^8^ cps at the sample position (High flux mode). The scattered X-rays were detected using a two-dimensional semiconductor detector (HyPix-6000) consisted of 765 × 813 pixels having the spatial resolution of 100 *μ*m. In these studies, the sample to detector distance of 1340 mm, with which the covered *Q*-range is 0.015 Å^−1^ < *Q* < 0.20 Å^−1^, was mainly used. For performing SEC-SAXS study on a small protein, the sample to detector distance of 350 mm, with which the covered *Q*-range is 0.035 Å^−1^ < *Q* < 0.60 Å^−1^, was also optioned.

### SAXS cell

We adopted a flat cell to avoid an interference scattering from the edge of capillary cell. This flat cell is made of SUS303. At the centre of SAXS cell, there is a sample space, which has a disk-like shape with a diameter of 2.5 mm and an optical path of 1 mm (volume: 4.9 *μ*l) and also has two quarts windows with 20 *μ*m thickness: X-ray irradiation zone, shown by black dotted circle (refer to Fig. 1). The transmission of empty SAXS cell was measured to 0.71 at the wavelength of 1.54 Å. The temperature at the SAXS cell is controlled by a peltier device.

### UV-SAXS cell

Deuterium UV light source is SL3 supplied from StellarNet Inc. It emits a deep UV spectral output over the 190–450 nm range and higher. It should be noted that SL3 is one of the brightest compact UV light sources with over 15 W/m^2^. As for a compact UV detector, Qmini2 UV supplied from RGB Photonics GmbH was used. It can evaluate for integrated UV - VIS - NIR ranging from 220 to 400 nm with the spectral resolution of 0.3 nm. The absorbance mode is utilized for our UV-SAXS-Cell system. At the upstream of SAXS-cell, UV source and compact UV detector are aligned via sample flow path on straight line (refer to Fig. 1). The sample flow path of UV-SAXS-cell was set to 5 mm and the time course of UV intensity is monitored by PC.

### Data reduction of SAXS data

Firstly, two-dimensional pattern comprised of 765 × 813 pixels are saved as 32-bit tiff format. Prior to data reduction, the beam centre and sample to detector distance are found with a scattering pattern of silver behenate using the software SAngler^15^. With the obtained beam centre and sample to detector distance, the two-dimensional scattering patterns are converted to a one-dimensional scattering profile by circular averaging. As a next step, one-dimensional scattering profile is corrected for transmittance by direct beam. In order to obtain the scattering profile of a protein in solution, the scattering profile of sample solution was subtracted from that of buffer. Then, the scattering intensity from the protein in solution is converted to absolute scale [cm^−1^] with the scattering intensity of water (*I*_water_ = 1.632 × 10^−2^ cm^−1^). All the procedures are performed by “SAngler” as well.

### HPLC system and SEC columns

Prominence (SHIMADZU) for HPLC, Superdex 200 increase 10/300 GL (GE Healthcare), Superdex 75 increase 10/300 GL (GE Healthcare) and Superdex 200 increase 3.2/300 GL (GE Healthcare) for a size exclusion columns were utilized for the present La-SSS, respectively.

### Samples

For testing the performance of La-SSS, we have prepared five protein solutions, PNGase-PUB, OVA, Fc, AF, and BSA solutions as representatives of the relatively lower, middle, also middle, higher molecular weight proteins and multi-component system, respectively. OVA, BSA and AF were purchased from Sigma Aldrich Co. LLC. and the catalogue number of OVA, AF and BSA are A7641-250MG, A3641-100MG and A4612-1G, respectively. OVA, AF and BSA were dissolved in 100 mM Tris/HCl (pH 7.5), 100 mM NaCl without any further purification. Preparations of Fc and PNGase-PUB proteins were performed according to methods in the previous papers^10,14^. The injected initial protein concentration of OVA, Fc, BSA, AF and PNGase-PUB was 5.0 mg/mL, 5.0 mg/mL, 5.4 mg/mL, 5.1 mg/mL and 2.7 mg/mL, respectively, and the injected volume was 500 *μ*l, 500 *μ*l, 500 *μ*l, 500 *μ*l and 300 *μ*l and the temperatures were kept at 25 °C. Under this condition, the average protein concentration at monomeric OVA, Fc, BSA, AF and PNGase-PUB is ranged from 0.79 mg/mL to 2.5 mg/mL.

### Analytical ultracentrifuge

To check the distributions of oligomers in BSA solution, analytical ultracentrifuge (AUC) experiments were performed at 60000 rpm at 25 °C with XL-I, Beckmann Colter. The distributions of sedimentation coefficient (*C*(*s*_20_)) were analyzed with SEDFIT^16^.

## Supplementary information

Supporting Materials

## Data Availability

The datasets generated and analyzed during the current study are available from the corresponding authors on reasonable request.
