# Supplementary material for: Newly developed Laboratory-based Size exclusion chromatography Small-angle x-ray scattering System (La-SSS)
Source: Sci Rep. 2019 Aug 30;9:12610. doi: 10.1038/s41598-019-48911-w (PMC6717197; doi:10.1038/s41598-019-48911-w)
Supplement: Supplementary file 1 — Supporting Materials [file 41598_2019_48911_MOESM1_ESM.pdf]

Supplementary Materials

Newly developed Laboratory-based Size exclusion chromatography Small-angle x-ray scattering System (La-SSS)

Rintaro Inoue[1*], Tatsuo Nakagawa[2], Ken Morishima[1], Nobuhiro Sato[1], Aya Okuda[1], Reiko Urade[1], Rina Yogo[3,4], Saeko Yanaka[3,4], Maho Yagi-Utsumi[3,4], Koichi Kato[3,4], Kazuki Omoto[5], Kazuki Ito[5] and Masaaki Sugiyama[1*]

[1]*Institute for Integrated Radiation and Nuclear Science, Kyoto University, Kumatori, Sennan-gun, Osaka 590-0494 JAPAN.*

[2]*Unisoku Co. Ltd., 3-4-2 Kasugano, Hirakata City, Osaka 573-0131 JAPAN.*

[3]*Exploratory Research Center on Life and Living Systems (ExCELLS) and Institute for Molecular Science (IMS),National Institutes of Natural Sciences, 5-1 Higashiyama, Myodaiji, Okazaki, 444-8787, JAPAN.*

[4]*Graduate School of Pharmaceutical Sciences, Nagoya City University, 3-1 Tanabe-dori, Mizuho-ku, Nagoya, Aichi, 467-8603, JAPAN.*

[5]*Rigaku Corp. 12-9-3 Matsubara, Akishima City, Tokyo 196-8666 JAPAN.*

*Corresponding authors. R. I. rintaro@rri.kyoto-u.ac.jp, M. S. sugiyama@rri.kyoto-u.ac.jp

This PDF file includes:

Fig. S1, Fig. S2, Fig. S3, Fig. S4 and Table S1.

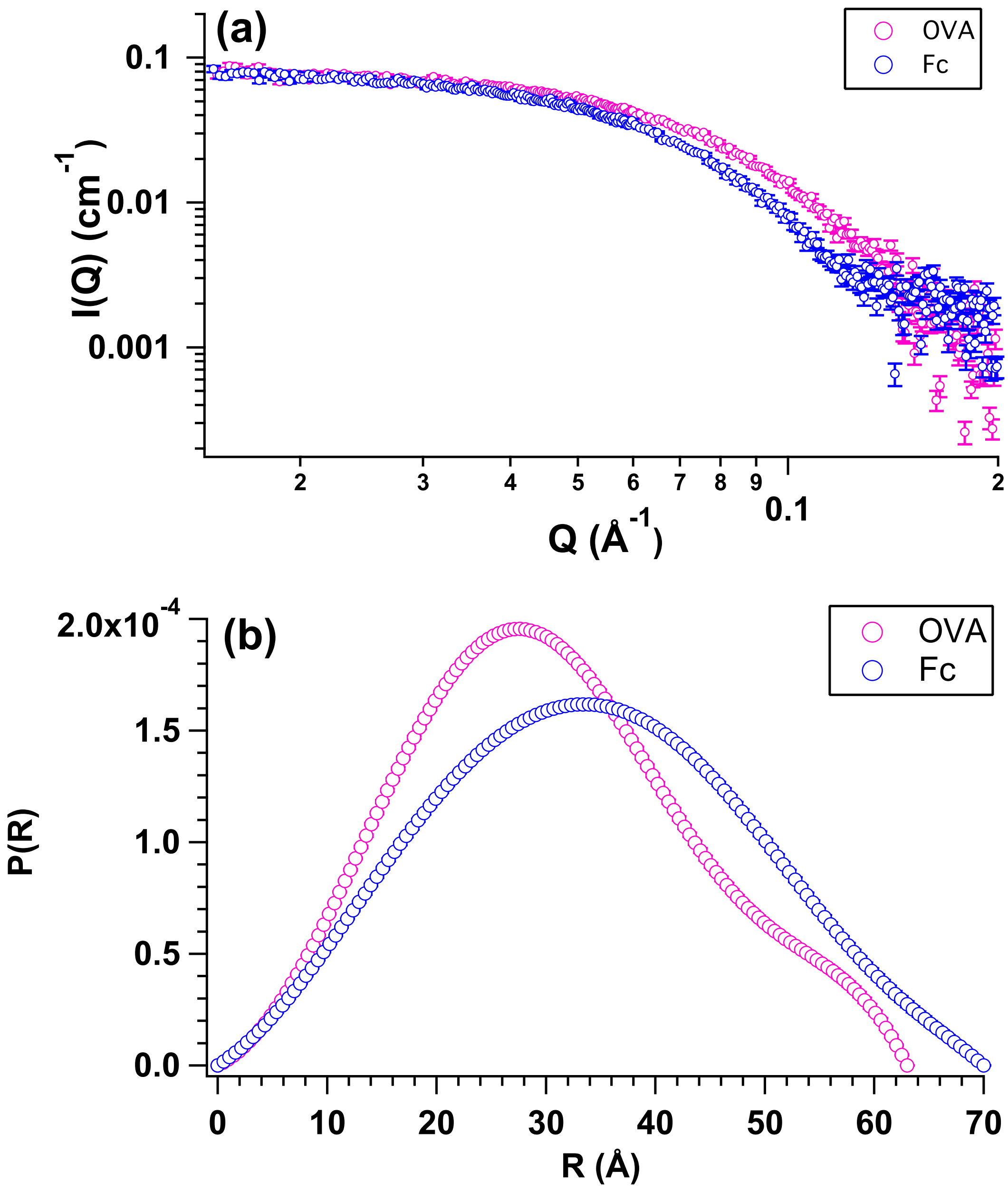

Fig. S1. (a) SAXS profiles of OVA and Fc. (b)*P*(*R*) functions of monomeric OVA and Fc.

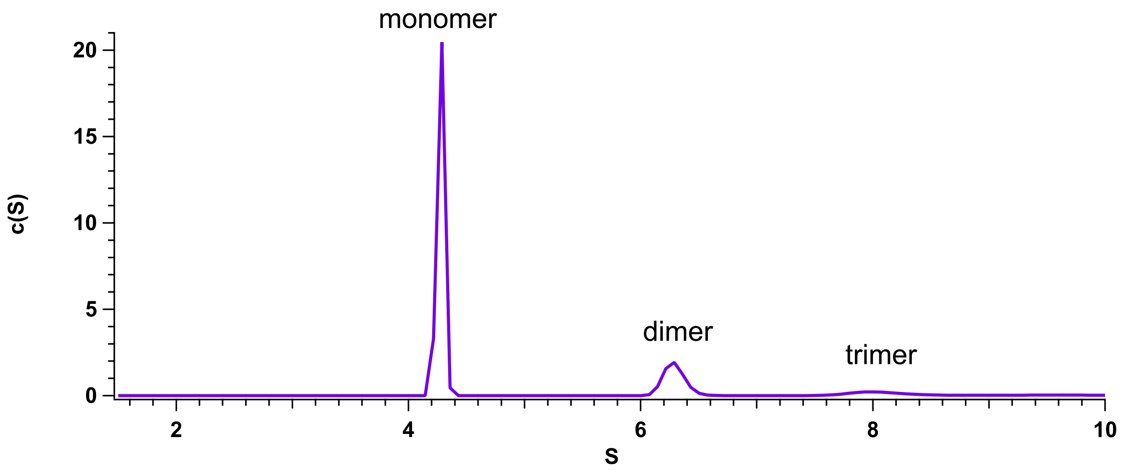

Fig. S2. The distribution of sedimentation coefficient (c(S_{20})) of BSA solution at the concentration of 5.4 mg/mL. The peaks at around S_{20}=4, S_{20}=6 and S_{20}=8 correspond to monomeric, dimeric and trimeric BSA oligomers, respectively.

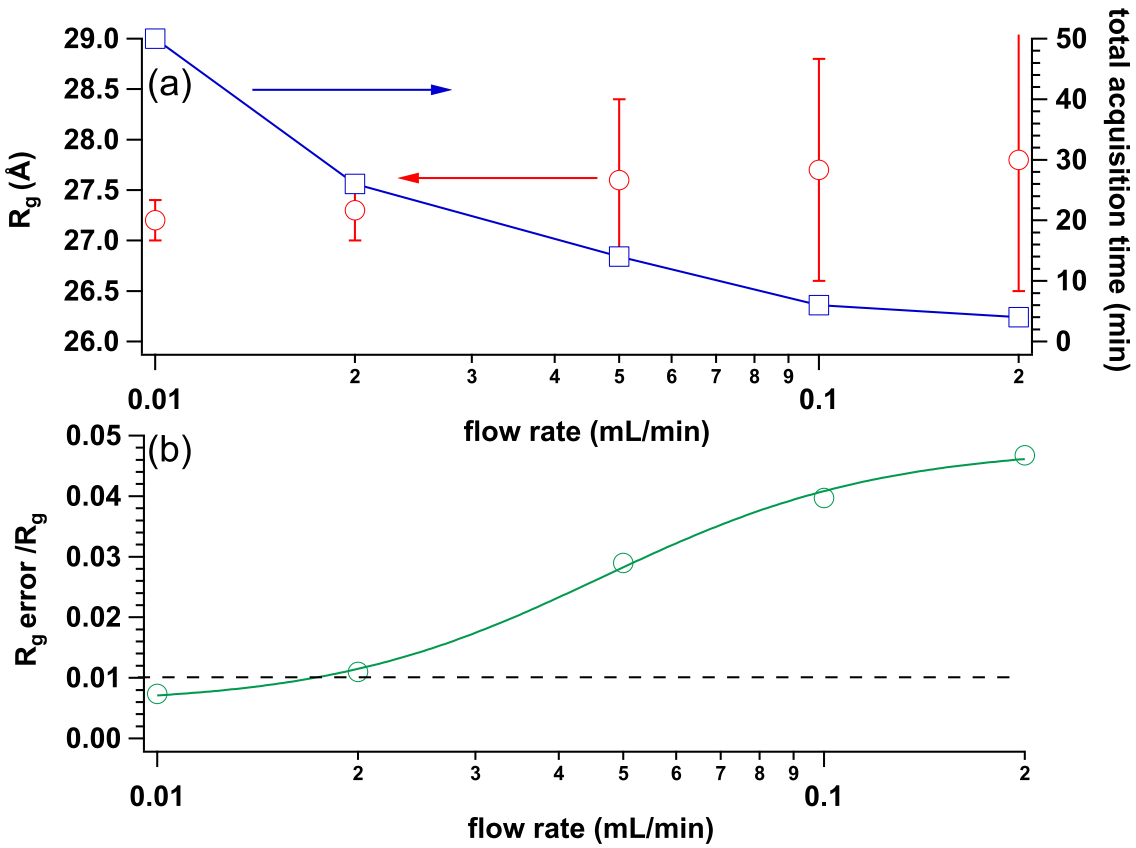

Fig. S3 (a) The dependence of R_g and total data acquisition time with flow rate. (b) The dependence of R_g error/R_g with flow rate and the solid curve was guided by eye.

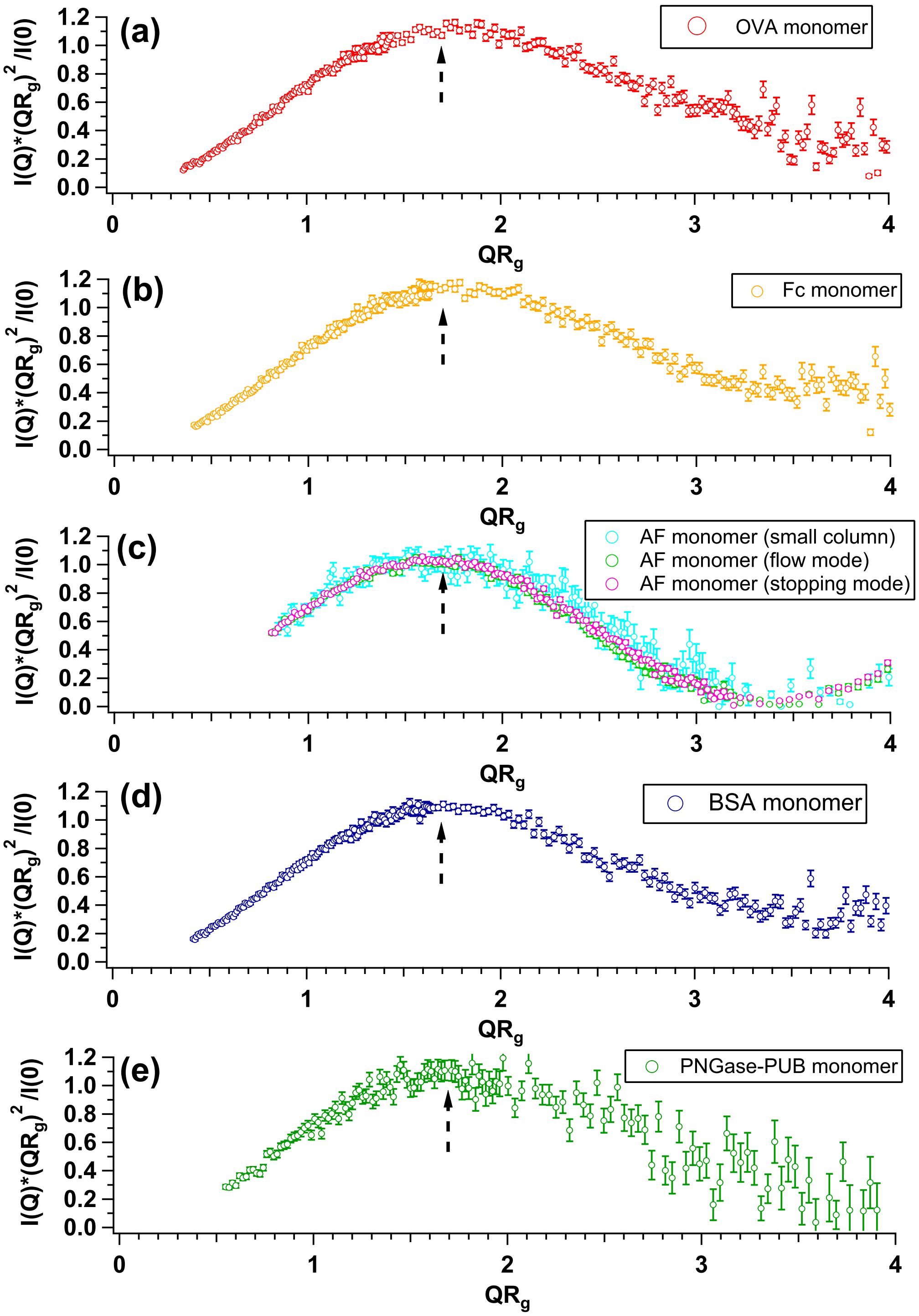

Fig. S4. Dimensionless Kratky plots of OVA monomer (a), Fc monomer (b), AF monomer (c), BSA monomer (d), PNGase-PUB monomer (e), respectivley. In the panel (c), light blue, light green and pink circles measured by the small system, flow mode and stopping mode, respectively. Dotted arrows correspond to QR_g=1.7.

Detailed steup for La-SSS	Products and Manufucturer
HPLC-components	Degasser (DGU-20A, SHIMADZU Co., Ltd.)
	Pump (LC-20Ai, SHIMADZU Co., Ltd.)
	Column oven (CTO-20AC, SHIMADZU Co., Ltd.)
	Contorl bus unit (CBM-20A, SHIMADZU Co., Ltd.)
	UV detector (SPD-20A, SHIMADZU Co., Ltd.)
	Fraction collector (FRC-10A, SHIMADZUCo., Ltd.)
auto valve	VF21-1005 (FLOM Corp.)
SAXS cell	Flow cell (Unisoku. Co., Ltd.)
SAXS cell UV source	Deuterium light source for UV, SL3 (StellarNet Inc.)
SAXS cell UV detector	Compact UV spectrometer, Qmini2 UV (RGB Photonics GmbH)
Tubing	PEEK 0.13 mm (ISIS ltd.)

Table S1. Specifications of products concerned for La-SSS.
